# Reasons for smoking relapse according to time since quit attempt

**DOI:** 10.36416/1806-3756/e20250208

**Published:** 2026-05-22

**Authors:** Aldo Agra de Albuquerque, Oliver A Nascimento, Lygia de Carvalho Sampaio, Talita Cepas Lobo, Rosangela Vicente dos Santos, José Roberto Jardim

**Affiliations:** 1. Núcleo de Prevenção e Cessação do Tabagismo - PrevFumo - Divisão de Pneumologia, Universidade Federal de São Paulo - Unifesp - São Paulo (SP) Brasil.; 2. Faculdade de Medicina São Leopoldo Mandic, Campinas (SP), Brasil.

**Keywords:** Recurrence, Smoking cessation, Tobacco use disorder, Risk factors

## Abstract

**Objective:**

To investigate the primary reasons for smoking relapse and examine how they vary according to the time elapsed between abstinence and relapse.

**Methods:**

This was a cross-sectional study analyzing 1,305 adult smokers who sought treatment at a smoking cessation center in Brazil between January of 2018 and December of 2020. All participants had previously attempted to quit smoking. On the basis of the longest duration of abstinence achieved, participants were divided into two groups: early relapse (within six months) and late relapse (after six months). The main reasons for relapse were assessed for the overall population and compared between the two groups.

**Results:**

The most frequently reported reasons for relapse were chemical dependence, anxiety, stress due to everyday problems, and irritability/anger. In comparison with individuals with late relapse, those with early relapse more commonly cited chemical dependence (45.0% vs. 20.5%; p < 0.001), anxiety (39.2% vs. 27.3%; p < 0.001), and irritability (20.5% vs. 13.4%; p = 0.001). In contrast, late relapse was more often associated with stress due to everyday problems (35.5% vs. 23.1%; p < 0.001), exposure to high-risk situations (19.6% vs. 9.2%; p < 0.001), and interacting with smokers outside the home (10.7% vs. 5.6%; p = 0.001).

**Conclusions:**

Reasons for smoking relapse vary over time. Early relapse is more frequently associated with chemical dependence, anxiety, and irritability, whereas late relapse is more commonly associated with stress, exposure to high-risk situations, and social exposure to smokers. These findings may guide tailored interventions based on the timing of relapse.

## INTRODUCTION

Smoking is considered a chronic disease characterized by periods of remission and relapse over time, with relapse constituting a natural part of the disease trajectory.^1^ Even with optimized treatment combining behavioral and pharmacological interventions, one-year abstinence rates remain at approximately 30%.^2^ ^,^ ^3^

The process preceding relapse is highly variable among smokers and across multiple quit attempts by the same individual. Similarly, the reasons that lead to relapse vary from person to person and may change throughout the life of an individual.^4^ The relapse process typically shows a high rate in the early months following cessation, followed by a progressive decline as abstinence duration increases. Most relapse episodes appear to occur within the first six months of cessation.^1^ ^,^ ^5^ ^,^ ^6^

The main factors associated with relapse include chemical dependence and withdrawal symptoms, as well as stress, anxiety, depressive symptoms, living with other smokers, exposure to smoking-related cues, and weight gain.^7^ ^-^ ^14^ These reasons for relapse appear to vary depending on the timing of the relapse. For example, craving and anxiety are more commonly associated with early relapse, whereas living with smokers and weight gain are more commonly associated with later relapse.^12^ ^,^ ^15^ ^,^ ^16^ However, these findings are not definitive, as many studies have involved small sample sizes, limited follow-up periods, and monitoring by smoking cessation technicians, which may introduce bias and fail to fully reflect real-life conditions. 

Understanding the reasons for relapse and their temporal patterns can improve our understanding of the relapse process and inform the selection of targeted interventions during treatment. The objective of the present study was to identify the main reasons for smoking relapse and their temporal distribution. 

## METHODS

This was a cross-sectional study based on a retrospective analysis of the database of patients treated between January of 2018 and December of 2020 at the Federal University of São Paulo Smoking Prevention and Cessation Center, located in the city of São Paulo, Brazil. The study protocol was approved by the Institutional Review Board of the Federal University of São Paulo *Hospital São Paulo*, also located in the city of São Paulo (Protocol no. 47676321.4.0000.5505). The institutional review board waived the requirement for written informed consent and authorized the use of a Data Use Agreement, with data anonymization being achieved by removing all personally identifiable information prior to analysis. Data were collected during the initial assessment of smokers at the outpatient clinic with a standardized form completed by a trained health care professional. 

### Inclusion criteria

The study included individuals > 18 years of age spontaneously seeking treatment at the clinic or referred to the clinic for smoking cessation. Eligible participants had made at least one serious attempt to quit smoking and were users of conventional cigarettes (with or without concurrent use of other tobacco products). A serious quit attempt was defined as one resulting in at least one day of abstinence.^17^ Smokers with incomplete data were excluded. For patients with more than one evaluation during the study period, only the most recent was included in the analysis. 

### Assessments

The following variables were assessed: sociodemographic data (sex, age, marital status, and level of education); smoking history; and comorbidities (COPD, asthma, myocardial infarction, gastric ulcer, cancer, depression, and other psychiatric disorders). In addition, the Charlson Comorbidity Index was calculated. 

Smoking history included age of smoking onset, average number of cigarettes smoked daily over a lifetime, smoking load in pack-years, level of nicotine dependence as assessed by the Fagerström Test for Nicotine Dependence, and previous quit attempts. Participants were divided into two groups based on the longest period of abstinence achieved in a previous attempt: early relapse (relapse within six months) and late relapse (relapse after six months). 

### Relapse questions

Participants were asked to identify the reasons for their relapse by responding to the following open-ended question: “Why did you go back to smoking?” They could report one or multiple reasons. Their responses were matched against a predefined list of possible reasons. If a response did not match an existing category, it was classified as “other” and described accordingly. The list had originally been developed from an evaluation of 300 patients at our clinic and was later reviewed and refined by our team of specialists in tobacco cessation, including pulmonologists and a psychologist. 

The predefined list of reasons for smoking relapse included the following: dependence/strong cravings; anxiety; irritability/anger; depressive symptoms/sadness; stress due to everyday problems (e.g., financial/family issues); exposure to high-risk situations (e.g., relapsing at a party or bar); weight gain; living with smokers in the home; interacting with smokers in social or work settings; and self-testing (e.g., smoking a single cigarette to “test control”). Two additional options were available: “do not know” and “other.” 

Consecutive sampling was used, meaning that all consecutive patients attending our outpatient smoking cessation clinic during the study period were included. 

Data were stored in a Research Electronic Data Capture database (REDCap; Vanderbilt University, Nashville, TN, USA), used under an agreement with the Federal University of São Paulo. A Smoking Prevention and Cessation Center form served as the basis for the database, which was anonymized before analysis. 

### Statistical analysis

Descriptive statistics for categorical variables were presented as absolute values and proportions. Continuous variables were expressed as mean ± standard deviation for normally distributed data and as median and interquartile range for non-normally distributed data. Reasons for smoking relapse and relapse timing were analyzed by using means and proportions. Associations between reasons for relapse and relapse timing (early vs. late) were evaluated by the chi-square test. A value of p < 0.05 was considered significant. All analyses were performed with the IBM SPSS statistical software package, version 21.0 for Windows (IBM Corporation, Armonk, NY, USA). 

## RESULTS

**Figure 1:**
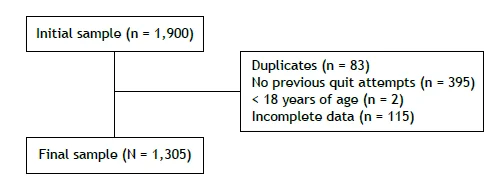
Flow chart of the study sample.

During the study period, 1,900 treatment records were identified. Of those, 83 were excluded for being duplicate entries, 395 were excluded because the individuals had never attempted to quit smoking, 2 were excluded because the individuals were < 18 years of age, and 115 were excluded because of incomplete data. The final sample comprised 1,305 individuals (Figure 1). 

The characteristics of the study population are presented in Table 1. Most (65%) of the study participants were women, with a mean age of 52.4 years. Most (74.8%) had an educational level of high school or less. The sample had a high cumulative smoking load and high levels of nicotine dependence. 

**Table 1:** Sociodemographic characteristics, smoking history, and comorbidities.^a^

Baseline characteristic	N = 1,305	
Female sex	855	(65.5%)
Age, years	52.4 ± 12.2	
Level of education		
Illiterate or incomplete elementary school	105	(8.0%)
Complete elementary school or incomplete middle school	179	(13.7%)
Complete middle school or incomplete high school	168	(12.9%)
Complete high school or incomplete college	525	(40.2%)
College (complete)	267	(20.5%)
Graduate school	61	(4.7%)
Marital status		
Single	383	(29.3%)
Married/in a stable union	543	(41.6%)
Divorced	183	(14.0%)
Separated	71	(5.4%)
Widowed	125	(9.6%)
Socioeconomic class*		
A	68	(5.2%)
B1	109	(8.4%)
B2	381	(29.2%)
C1	387	(29.7%)
C2	280	(21.5%)
D-E	80	(6.1%)
Age at smoking onset, years	15 [14-18]	
Smoking load, pack-years	39.9 ± 27.6	
Chemical dependence**	6 [4-7]	
Current number of cigarettes	19.3 ± 11.5	
Number of previous attempts to quit	2 [1-4]	
Longest duration of the previous attempt to quit		
Up to one week	254	(19.5%)
Over one week up to one month	212	(16.2%)
Over one month up to six months	318	(24.4%)
Over six months up to one year	222	(17.0%)
Over one year	299	(22.9%)
Charlson Comorbidity Index	1 [0-3]	
Self-reported comorbidities		
COPD	241	(18.5%)
Asthma	289	(22.1%)
Cerebrovascular disease	60	(4.60%)
Acute myocardial infarction	99	(7.6%)
Gastric ulcer	105	(8.0%)
Depression	459	(35.2%)
Other psychiatric disorders	191	(14.6%)
Neoplasia	119	(9.1%)

a Data expressed as n (%), mean ± SD, or median [IQR]. *A-B2: higher classes; C1 and C2: middle classes; and D and E: lower classes. **As assessed by the Fagerström Test for Nicotine Dependence.

The most commonly reported reasons for smoking relapse were chemical dependence, anxiety, stress due to everyday problems, and irritability/anger (Table 2). The median number of reasons cited per individual was 1 (IQR, 1-2). 

**Table 2:** Reasons for smoking relapse by time of relapse.

	Early		Late			
Reason for smoking relapse	n = 623	%	n = 451	%	p*	OR
Dependence/strong cravings	353	45.0%	107	20.5%	< 0.001	4.2 (3.21-5.5)
Anxiety	307	39.2%	142	27.3%	< 0.001	2.11(1.64-2.72)
Stress due to everyday problems	181	23.1%	185	35.5%	< 0.001	0.59 (0.46-0.76)
Irritability/anger	161	20.5%	70	13.4%	0.001	1.90 (1.39-2.59)
Depressive symptoms/sadness	90	11.5%	69	13.2%	0.340	0.93 (0.67-1.31)
Being exposed to a situation that is considered risky	72	9.2%	102	19.6%	< 0.001	0.45 (0.32-0.62)
Weight gain	14	1.8%	14	2.7%	0.271	0.72 (0.34-1.52)
Living with smokers in the home	41	5.2%	30	5.8%	0.680	0.99 (0.61-1.61)
Interacting with smokers outside the home	44	5.6%	56	10.7%	0.001	0.54 (0.35-0.81)
Test oneself	25	3.2%	22	4.2%	0.326	0.82 (0.45-1.47)
Does not know	18	2.3%	18	3.5%	0.211	0.72 (0.37-1.39)
Other	64	8.2%	51	9.8%	0.310	0.90 (0.61-1.33)

*Chi-square test.

Among those with early relapse (within six months), the most frequently reported reasons were chemical dependence, anxiety, stress due to everyday problems, and irritability. In contrast, individuals with late relapse (after six months) most commonly reported stress due to everyday problems, anxiety, chemical dependence, and exposure to high-risk situations. 

**Figure 2:**
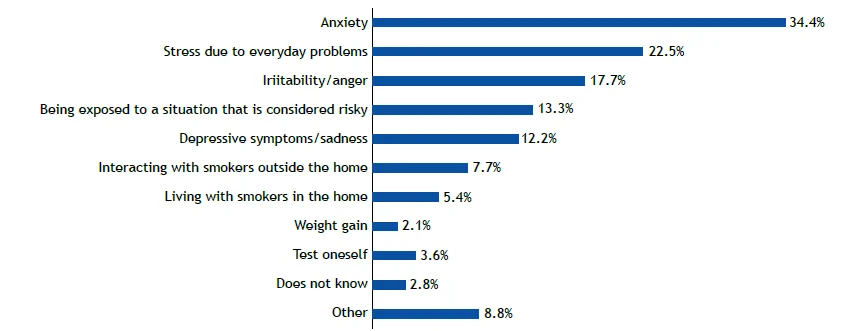
Frequency of reasons for smoking relapse.

When we compared the distribution of reasons by time of relapse, several differences were observed (Figure 2). Smokers who relapsed early more frequently reported dependence, anxiety, and irritability, whereas those who relapsed later more commonly cited stress due to everyday problems, exposure to high-risk situations, and living with smokers or interacting with smokers outside the home. 

## DISCUSSION

The present study showed that the main reasons for smoking relapse included dependence, anxiety, stress due to everyday problems and irritability, and that when the time of relapse was taken into consideration, early relapse was associated with chemical dependence, anxiety, and irritability, whereas late relapse was associated with chemical dependence, anxiety, stress due to everyday problems, exposure to high-risk situations, and interacting with smokers outside the home. The main reasons for smoking relapse found in the present study are consistent with those described in the literature, i.e., dependence, anxiety, and stress.^7^ ^-^ ^14^

In a small study involving 62 former smokers in Brazil, craving (in 14%) and stress (in 14%) were the main reasons for relapse,^8^ a finding that is consistent with those of the present study. However, the authors of the aforementioned study^8^ did not take into account the time to relapse. Brown et al.^14^ investigated a small number of North American smokers (N = 60) and found an increased risk of relapse in the first week, being significantly associated with anxiety and pointing out to a possible relationship between anxiety and a higher expectation of negative effects on mood as a result of smoking cessation. Anxiety was also an important factor in our study, being the second main cause of relapse (in 34.4%). Buczkowski et al.^9^ reported that stress is one of the main reasons for relapse and cited the additional risk of exposure to smokers as another reason for relapse. Torres et al.^13^ also reported that stress is one of the main causes of relapse and that women use tobacco more as a strategy for coping with stress than do men. 

In a study of a large number of smokers in Denmark (N = 2,621), the main causes of relapse were as follows: missing smoking at parties (in 74.3%) and in everyday life (in 74%); having family and friends who smoke (in 58.6%); nervousness, agitation, and feeling depressed (in 49.4%); weight gain (in 40.8%); and something important that had happened (in 33%).^10^ It is quite possible that cultural factors might play an important role in smoking relapse, given that in our study only 7.7% and 5.4% of the participants reported that interacting with smokers outside the home and living with smokers in the home were the causes of relapse, respectively; another reason that contrasted with those reported in the Danish study^10^ was that only 2.1% of our smokers claimed weight gain as a reason to start smoking again. The high relapse rate associated with activities related to happy moments, such as partying, is of note; in one study, however, only 17% of relapses were reported to happen during happy moments.^18^ Irfan et al.^19^ evaluated the reasons for smoking relapse in a predominantly male population and found data that are similar to those of our study, with the first cause of relapse being dependence (in 15.2%), followed by peer pressure (in 12%), anxiety (in 5.3%), and stress and mood changes (in 3%). 

The differences in reasons for smoking relapse across studies are most likely due to the heterogeneity of the methods employed and the sizes of the samples. Cultural factors prevalent in each country and individual motivation to quit smoking might also play a role. It is possible that stronger reasons such as personal or family illness or setting an example for children or grandchildren could influence the relapse rate, as could religion, the price of cigarettes, and government actions. 

When we compared the early and late relapse groups, we found that some of the reasons for smoking relapse were common and important in both groups, such as dependence, anxiety, and stress. However, other reasons reported by the study participants show important differences between the early and late relapse groups. Chemical dependence, anxiety, and irritability were found to be more important among those in the early relapse group, being importantly related to withdrawal syndrome, whereas, in the late relapse group, exposure to high-risk situations, stress due to everyday problems, and interacting with smokers outside the home were the most common reasons for smoking relapse. 

Brown et al.^14^ found an association between anxiety and higher relapse rates in the first week, showing a relationship between chemical dependence and relapse in the first moments after cessation. Despite evaluating relapse in the first week only, the aforementioned study^14^ supports our findings of chemical dependence and anxiety being associated with early relapse. 

Our findings are in agreement with those of a study by Nøorregaard et al.^15^, in which craving and irritability were the main reasons for early relapse, whereas external causes (social order and other problems) were more closely associated with late relapse, supporting the hypothesis that the reasons for smoking relapse are quite different in relation to time.^15^

A novel finding of the present study is that there is a clear difference in the reasons for early or later relapse during the process of smoking cessation. Chemical dependence is a major influence on relapse at the early stages of smoking cessation, whereas reasons such as stress due to everyday problems, exposure to high-risk situations, and living with smokers become more important as the time of abstinence increases. 

Our findings are very important to clinical practice because they help to understand the process of smoking relapse in relation to time and may influence the treatment of smokers by optimizing management on the basis of the point at which each smoker is in the smoking cessation process. On the other hand, because the reasons for relapse might differ across cultures, further studies are needed in order to evaluate the influence of a local culture on the smoking cessation process. 

The present study has some limitations. First, the study population was predominantly female, which is different from the general smoking population, most of whom are male. However, this is consistent with what is seen in most health services in Brazil, where women seek health services more often and take better care of their own health than do men. Therefore, the higher number of women in our study might be a consequence of a greater concern with health on the part of women in Brazil. Because this was not the objective of the present study, we did not test this hypothesis.^20^ Second, the study population might not correspond to the general population of smokers because the study participants were selected from a referral center for smoking cessation, possibly showing a higher degree of cigarette dependence than that seen in the general population of smokers. However, this was a very interesting population to be studied because, in addition to high cigarette dependence, they had all made unsuccessful attempts at quitting and experienced greater difficulty in doing so. This is a population of patients that need help and better understanding because they challenge our current knowledge of the smoking cessation process. A third limitation of the present study is that much of the information collected depended on the memory of the participants, which may partially impair the quality of the data. However, if we were to prospectively evaluate a population of smokers, we would need a long period of time to observe all the possibilities that the present study allowed us to evaluate, such as pregnancy and hospitalization, which are not events that occur frequently during life. A fourth limitation is that we did not individually evaluate the quit attempt of each participant globally. We may therefore have failed to interpret nuances of each attempt, especially in relation to time, which was the focus of the present study; however, by generalizing previous attempts we were able to analyze the main points associated with relapse. 

To our knowledge, this was the first study in Brazil involving a large number of smokers and assessing the time to relapse with the objective of studying the reasons for smoking relapse. Recognizing that the reasons for relapse vary over the course of the smoking cessation process is crucial for optimizing treatment strategies. Our findings indicate that relapse within the first six months is more frequently associated with chemical dependence, anxiety, and irritability, whereas relapse occurring after six months is more commonly associated with stress, exposure to high-risk situations, and interacting with smokers outside the home. These insights can contribute to the development of more targeted and time-sensitive interventions, enhancing the effectiveness of smoking cessation programs. 

## References

[1] Clinical Practice Guideline Treating Tobacco Use and Dependence 2008 Update Panel.Liaisons.and Staff (2008). A clinical practice guideline for treating tobacco use and dependence 2008 update. A U.S. Public Health Service report. Am J Prev Med.

[2] Tobacco Use and Dependence Guideline Panel (2008). Treating Tobacco Use and Dependence: 2008 Update.

[3] Babb S, Malarcher A, Schauer G, Asman K, Jamal A (2017). Quitting Smoking Among Adults - United States, 2000-2015. MMWR Morb Mortal Wkly Rep.

[4] Shiffman S, Hickcox M, Paty JA, Gnys M, Kassel JD, Richards TJ (1996). Progression from a smoking lapse to relapse Prediction from abstinence violation effects, nicotine dependence, and lapse characteristics. J Consult Clin Psychol.

[5] Garvey AJ, Bliss RE, Hitchcock JL, Heinold JW, Rosner B (1992). Predictors of smoking relapse among self-quitters a report from the Normative Aging Study. Addict Behav.

[6] Barua RS, Rigotti NA, Benowitz NL, Cummings KM, Jazayeri MA, Morris PB (2018). 2018 ACC Expert Consensus Decision Pathway on Tobacco Cessation Treatment A Report of the American College of Cardiology Task Force on Clinical Expert Consensus Documents. J Am Coll Cardiol.

[7] Peuker AC, Bizarro L (2015). Características do processo de cessação do tabagismo na abstinência prolongada [Article in Portuguese] Context. Clin.

[8] Jesus MC, Silva MH, Cordeiro SM, Kortchmar E, Zampier VS, Merighi MA (2016). Understanding unsuccessful attempts to quit smoking a social phenomenology approach [Article in Portuguese]. Rev Esc Enferm. USP.

[9] Buczkowski K, Marcinowicz L, Czachowski S, Piszczek E (2014). Motivations toward smoking cessation, reasons for relapse, and modes of quitting Results from a qualitative study among former and current smokers. Patient Prefer Adherence.

[10] Pisinger C, Aadahl M, Toft U, Jørgensen T (2011). Motives to quit smoking and reasons to relapse differ by socioeconomic status. Prev Med.

[11] Koçak ND, Eren A, Boga S, Aktürk ÜA, Öztürk ÜA, Arinç S (2015). Relapse rate and factors related to relapse in a 1-year follow-up of subjects participating in a smoking cessation program. Respir Care.

[12] Ward KD, Klesges RC, Zbikowski SM, Bliss RE, Garvey AJ (1997). Gender differences in the outcome of an unaided smoking cessation attempt. Addict Behav.

[13] Torres OV, O'Dell LE (2016). Stress is a principal factor that promotes tobacco use in females. Prog Neuropsychopharmacol Biol Psychiatry.

[14] Brown RA, Kahler CW, Zvolensky MJ, Lejuez CW, Ramsey SE (2001). Anxiety sensitivity Relationship to negative affect smoking and smoking cessation in smokers with past major depressive disorder. Addict Behav.

[15] Nøorregaard J, Tønnesen P, Petersen L (1993). Predictors and reasons for relapse in smoking cessation with nicotine and placebo patches. Prev Med.

[16] Park EY, Lim MK, Kim BM, Jeong BY, Oh JK, Yun EH (2015). Factors Related to Relapse After 6 Months of Smoking Cessation Among Men in the Republic of Korea A Cross-Sectional Study. Medicine (Baltimore).

[17] Centers for Disease Control. Center for Health Statistics DRAFT Topics under Consideration for Redesigned National Health Interview Survey (NHIS) Sample Adult Questionnaire.

[18] Cummings KM, Jaén CR, Giovino G (1985). Circumstances surrounding relapse in a group of recent exsmokers. Prev Med.

[19] Irfan M, Haque AS, Shahzad H, Samani ZA, Awan S, Khan JA (2016). Reasons for failure to quit A cross-sectional survey of tobacco use in major cities in Pakistan. Int J Tuberc Lung Dis.

[20] Travassos C, Viacava F, Pinheiro R, Brito A (2002). Utilization of health care services in Brazil gender, family characteristics, and social status [Article in Portuguese]. Rev Panam Salud. Publica.

